# Low serum interleukin‐38 levels in patients with Graves’ disease and Hashimoto’s thyroiditis

**DOI:** 10.1002/jcla.24101

**Published:** 2021-11-19

**Authors:** Jialu Xu, Guoqing Huang, Linjie Weng, Luping Gong, Yushan Mao, Yan Li, Mingcai Li

**Affiliations:** ^1^ Department of Clinical Laboratory The Affiliated Lihuili Hospital, Ningbo University Ningbo China; ^2^ Department of Immunology, Zhejiang Key Laboratory of Pathophysiology Ningbo University School of Medicine Ningbo China; ^3^ Department of Endocrinology, The Affiliated Hospital of Medical School Ningbo University Ningbo China

**Keywords:** autoimmune thyroid disease, C‐reactive protein, Graves’ disease, Hashimoto's thyroiditis, interleukin‐38

## Abstract

**Background:**

Autoimmune thyroid disease (AITD) mainly includes Graves’ disease (GD) and Hashimoto's thyroiditis (HT), which is caused by individual genetics, autoimmune dysfunction, and a variety of external environmental factors. Interleukin (IL)‐38 is involved in a wide range of autoimmune diseases, but little is known about IL‐38 expression in AITD.

**Methods:**

Fifty patients with GD, 50 with HT, and 50 healthy controls (HC) were enrolled in this study. Basic information of the participants was obtained through a physical examination. Immunological data were obtained by an automatic chemiluminescence immunoanalyzer. C‐reactive protein (CRP) concentrations and the white blood cell count were measured. Serum IL‐38 levels were determined by an enzyme‐linked immunosorbent assay.

**Results:**

Serum IL‐38 levels were significantly lower in the GD and HT groups than in the HC group (both *p* < 0.01). Serum CRP concentrations were significantly lower in the HT group than in the HC group (*p* < 0.05). Receiver operating characteristic curve analysis showed that the area under the curve was 0.7736 (*p* < 0.01) for IL‐38 and 0.7972 (*p* < 0.01) for IL‐38 combined with CRP in the GD group. In the HT group, the area under the curve was 0.7276 (*p* < 0.01) for IL‐38 and 0.7300 for IL‐38 combined with CRP (*p* < 0.01).

**Conclusions:**

The results suggest that serum IL‐38 level is a potential new diagnostic biomarker in patients with GD and HT.

## Graphical abstract

IL‐38 concentrations were reduced in the Hashimoto's thyroiditis (HT) and Graves’ disease (GD) groups compared with those in the healthy control group (A). In the GD group, serum IL‐38 concentrations were positively correlated with C‐reactive protein (CRP) concentrations (B) and the white blood cell count (WBC) (C). We evaluated the diagnostic value of IL‐38 concentrations and IL‐38 combined with CRP concentrations for autoimmune thyroid disease using receiver operating characteristic (ROC) curves. The area under curve (AUC) was 0.7276 using IL‐38 concentrations for the diagnosis of HT (D) and 0.7300 using IL‐38 concentrations in combination with CRP concentrations (E). The AUC was 0.7736 (F) when IL‐38 concentrations were used to diagnose GD and 0.7972 (G) when IL‐38 concentrations were combined with CRP concentrations.

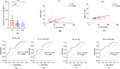

## 1 INTRODUCTION

Hyperthyroidism is a group of clinical syndromes due to excessive synthesis and secretion of thyroid hormones. Hyperthyroidism manifests clinically as palpitations, sweating, increased eating and bowel movements, and weight loss caused by hypermetabolism and sympathetic hyperexcitability. Graves’ disease (GD) is the leading cause of hyperthyroidism and characterized by decreased serum thyroid‐stimulating hormone (TSH) levels, increased serum thyroxine levels, and the persistence of TSH‐receptor antibody (TRAb).^1^ It has been demonstrated that GD is an autoimmune disease caused by a complex interaction between genetic and environmental factors.^2^ Some studies show a 3% risk of GD in women and 0.5% in men throughout the lifespan.^3^ The current treatment options for GD are mainly based on antithyroid medications and surgical intervention, but they have some limitations. Medication for GD leads to a high recurrence rate, while surgery results in the removal of thyroid tissue (with the risk of secondary hypothyroidism). Although the pathogenesis of GD is still not fully determined, some reports have shown that imbalance of cytokine expression levels in vivo plays a key role in the pathogenesis of the disease.^1^ In addition, some studies show that antithyroid drugs can reduce the production of thyroid pro‐inflammatory cytokines.^4^ An increasing number of studies have suggested that pro‐inflammatory cytokines, such as interleukin (IL)‐35, IL‐29, IL‐27, and tumor necrosis factor (TNF)‐α, may be involved in the development of GD.^5^, ^6^, ^7^, ^8^

Hashimoto's thyroiditis (HT) is an important component of autoimmune thyroid disease (AITD). HT is also an important cause of hypothyroidism and pathologically characterized by diffuse parenchymal atrophy, lymphocytic infiltration, and fibrosis.^9^ In addition, patients with HT are at higher risk of being affected by cardiovascular disease.^9^, ^10^ Currently, the incidence of HT is 0.3–1.5 cases per 1000 people^9^; the affected population is predominantly female, and the incidence in men is one‐tenth of that in women.^9^, ^11^ At present, there is no particularly good treatment for HT, and hypothyroidism is mainly controlled by hormone replacement (oral Levo‐Thyroxine4). Although the exact etiology of HT has not yet been fully clarified, cellular and humoral immunity plays a key role in the development of this disease. Evidence suggests that serum cytokines, such as IL‐6, TNF‐α, IL‐10, IL‐17, and IL‐22, play an important role in the pathogenesis of HT.^12^, ^13^, ^14^

IL‐38, which is a member of the IL‐1 family, is highly homologous to IL‐1 receptor antagonist (IL‐1Ra) and IL‐36Ra, and is functionally an anti‐inflammatory cytokine.^15^ IL‐38 is mainly expressed in the placenta, heart, liver, thymus, spleen, and fetal tissue.^15^ A significant association has been reported between IL‐38 levels and autoimmune diseases, such as rheumatoid arthritis (RA),^16^ systemic lupus erythematosus (SLE),^17^ and Behçet's disease.^18^

On the basis of the above‐mentioned evidence on the role of IL‐38 in autoimmune diseases and the paucity of relevant data in AITD studies, we aimed to investigate serum IL‐38 expression in patients with AITD and its relationship with corresponding antibodies.

## 2 MATERIAL AND METHODS

### 2.1 Research subjects

The subjects in this study were divided into the GD group, HT group, and healthy control (HC) group, with 50 people in each group. All participants were outpatients or inpatients at the Affiliated Lihuili Hospital, Ningbo University, between March 2020 and May 2021. The participants signed an informed consent form. The experimental protocol was approved by the Ethics Committee of the Affiliated Lihuili Hospital, Ningbo University.

The diagnostic criteria for GD were as follows: (1) hypermetabolic symptoms and signs; (2) diffuse goiter; (3) biochemical indices, which comprised decreased TSH concentrations, increased free triiodothyronine 4 (FT4) and serum total thyroxine 4 concentrations, and positive serum TRAb or thyroid peroxidase antibody (TPOAb); (4) ocular protrusion and other infiltrative eye signs; and (5) anterior tibial mucinous edema. The diagnostic criteria for HT were as follows: (1) diffuse enlargement of the thyroid gland; (2) positive antithyroglobulin antibody (TgAb) and TPOAb; and (3) a thyroid puncture biopsy consistent with fine‐needle aspiration cytological changes.

The inclusion criteria were as follows: subjects in the GD group met the diagnostic criteria of GD; subjects in the HT group met the diagnostic criteria of HT; and all participants were aged ≥18 years. The exclusion criteria were as follows: pregnancy, hypoproteinemia, hormone administration, infection, trauma, and other autoimmune diseases.

### 2.2 Biochemical parameter measurements

All subjects fasted overnight before blood collection, and blood samples were collected from 8:00 to 9:00 a.m. on the following day. Blood samples were stored at room temperature for 2 h and centrifuged at 1000 × *g* for 20 min, and the supernatant was collected and stored in sterile enzyme‐free tubes at −80°C.

TRAb was detected using a fully automated chemiluminescent immunoassay analyzer (MAGLUMI4000plus; Shenzhen New Industries). FT3, FT4, TSH, TgAb, and TPOAb were measured using a fully automated chemiluminescent immunoassay analyzer (i‐2000; Abbott). Biochemical parameters, such as the white blood cell count (WBC) and C‐reactive protein (CRP) concentrations, were measured by an automatic biochemical analyzer (AU5800; Beckman). All measurements were carried out in strict accordance with the equipment operating procedures.

### 2.3 IL‐38 measurement

Enzyme‐linked immunosorbent assays (ELISA) were used to measure serum IL‐38 protein concentrations in all participants. These ELISA kits were obtained from Shanghai Jianglai Biotechnology Co., Ltd. The experimental procedure was performed in strict accordance with the manufacturer's instructions. All serum samples were tested in duplicate. The same numbers of serum samples from different groups were randomly selected for testing in the same analytical batch.

### 2.4 Statistical analysis

Statistical analysis and creation of graphs were performed using GraphPad Prism 8.0 (GraphPad Software Inc.). Measurement data are expressed as mean ± standard deviation (SD). Count data are expressed as the percentage. For the measurement data, one‐way analysis of variance was used for comparisons between the three groups, and the independent samples *t* test was used for comparisons between two groups. Comparisons between the count data were performed using the chi‐squared test. Principal component analysis was used to assess the ability of IL‐38, CRP, WBC, and thyroid‐related parameters to classify all samples in the three groups. Pearson correlation analysis was performed to assess the correlation between IL‐38, CRP, and thyroid‐related parameters. *p* < 0.05 was considered to be statistically significant.

## 3 RESULTS

### 3.1 Clinical and biochemical parameter data

Table 1 shows the basic clinical information and biochemical parameters of the participants in the HC, HT, and GD groups. The mean age of participants and the proportion of women in the HT group were significantly higher compared with those in the HC group (both *p* < 0.05). The remaining biochemical parameters related to AITD were consistent with the characteristic manifestations of HT and GD.

**TABLE 1:** Basic clinical information and biochemical parameters

Variables	HC group (*n* = 50)	HT group (*n* = 50)	GD group (*n* = 50)	F/χ^2^	*p*‐value
Age (years)	36.4 ± 11.8	43.8 ± 15.0^*^	37.3 ± 9.7	4.285	0.0155
Gender (female, %)	68.0	88.0^*^	60.0^#^	10.32	0.0057
FT3 (pmol/L)	4.4 ± 0.4	4.1 ± 0.6	12.6 ± 8.5^*,#^	47.78	<0.0001
FT4 (pmol/L)	12.8 ± 1.0	12.6 ± 2.0	24.8 ± 7.7^*,#^	113.4	<0.0001
TSH (mIU/L)	1.8 ± 0.9	6.3 ± 8.1^*^	0.003 ± 0.007^#^	23.98	<0.0001
TgAb (IU/ml)	1.1 ± 0.7	799.2 ± 1439.0^*^	322.1 ± 597.9^#^	9.957	<0.0001
TPOAb (IU/ml)	0.3 ± 0.2	930 ± 1006.0^*^	846.8 ± 971.0^*^	20.31	<0.0001
TRAb (IU/ml)	0.4 ± 0.2	0.4 ± 0.4	15.2 ± 16.4^*,#^	40.85	<0.0001

Abbreviations: #significant compared with the HT group; FT3, free triiodothyronine 3; FT4, free triiodothyronine 4; GD, Graves’ disease; HC, healthy control; HT, Hashimoto's thyroiditis; TgAb, antithyroglobulin antibody; TPOAb, thyroid peroxidase antibody; TRAb, TSH‐receptor antibody. ^*^Significant compared with the HC group; TSH, thyroid‐stimulating hormone.

### 3.2 Serum IL‐38 and CRP concentrations, and the WBC count in AITD

**FIGURE 1:**
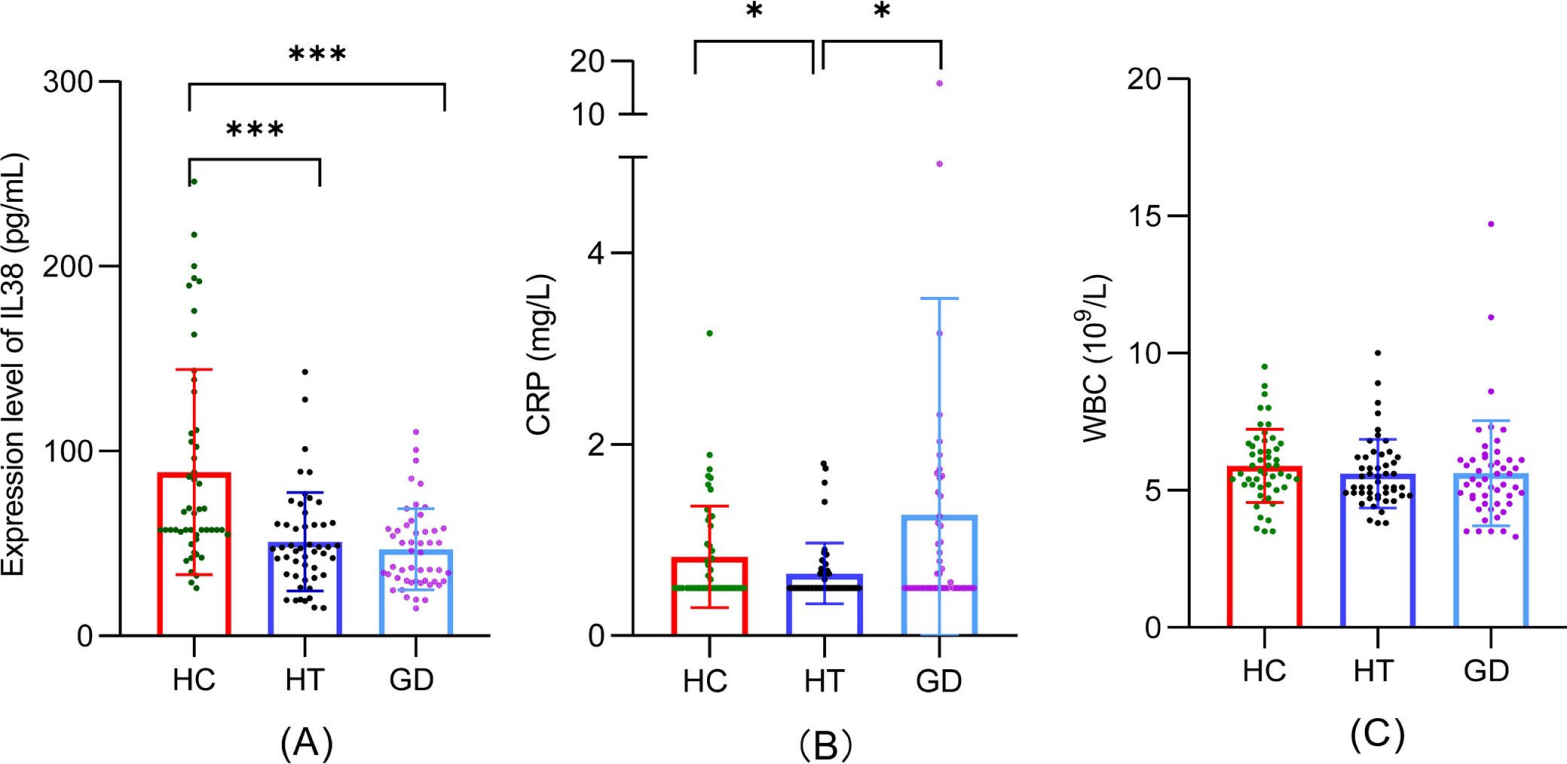
Serum IL‐38 concentrations, CRP concentrations, and the WBC count in AITD. (A) IL‐38 mean concentration in the HC group was 88.7 ± 55.5 pg/ml, those in the HT group was 51.0 ± 26.6 g/ml, and those in the GD group was 46.9 ± 21.9 pg/ml. (B) CRP mean concentrations in the HC group was 0.8 ± 0.5 mg/L, those in the HT group was 0.6 ± 0.3 mg/L, and those in the GD group were 1.3 ± 2.3 mg/L. (C)The mean WBC count in the HC group was (5.9 ± 1.3) × 10^9^/L; that in the HT group was (5.6 ± 1.2) × 10^9^/L, and that in the GD group was (5.6 ± 1.9) × 10^9^/L. HC, healthy control; HT, Hashimoto's thyroiditis; GD, Graves’ disease; CRP, C‐reactive protein; WBC, white blood cell

We found that IL‐38 concentrations were reduced in the HT (*p* < 0.01) and GD (*p* < 0.01) groups compared with those in the HC group (Figure 1A). The HT group had lower CRP concentrations than those in the HC group (*p* < 0.05), while the GD group had higher CRP concentrations than those in the HT group (*p* < 0.05) (Figure 1B). CRP concentrations tended to be higher in the GD group than in the HC group, but this was not significant. The WBC count was not significantly different between the three groups (Figure 1C).

### 3.3 Principal component analysis

**FIGURE 2:**
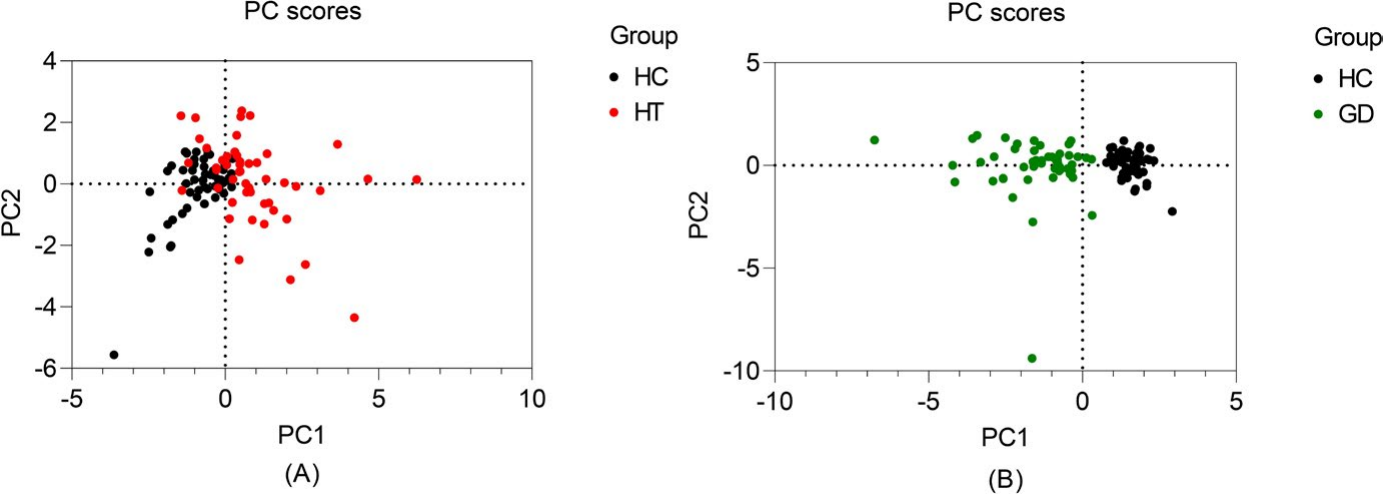
Principal component analysis score plot. Black circles represent individuals in the HC group, red circles represent individuals in the HT group, and green circles represent individuals in the GD group

We performed principal component analysis in the three groups of participants by combining IL‐38, CRP, WBC, and other parameters reflecting thyroid function. These indicators were able to separate HT and GD from HC (Figure 2A,B).

### 3.4 Correlation analysis between serum IL‐38 concentrations, inflammatory markers, and thyroid function parameters

**FIGURE 3:**
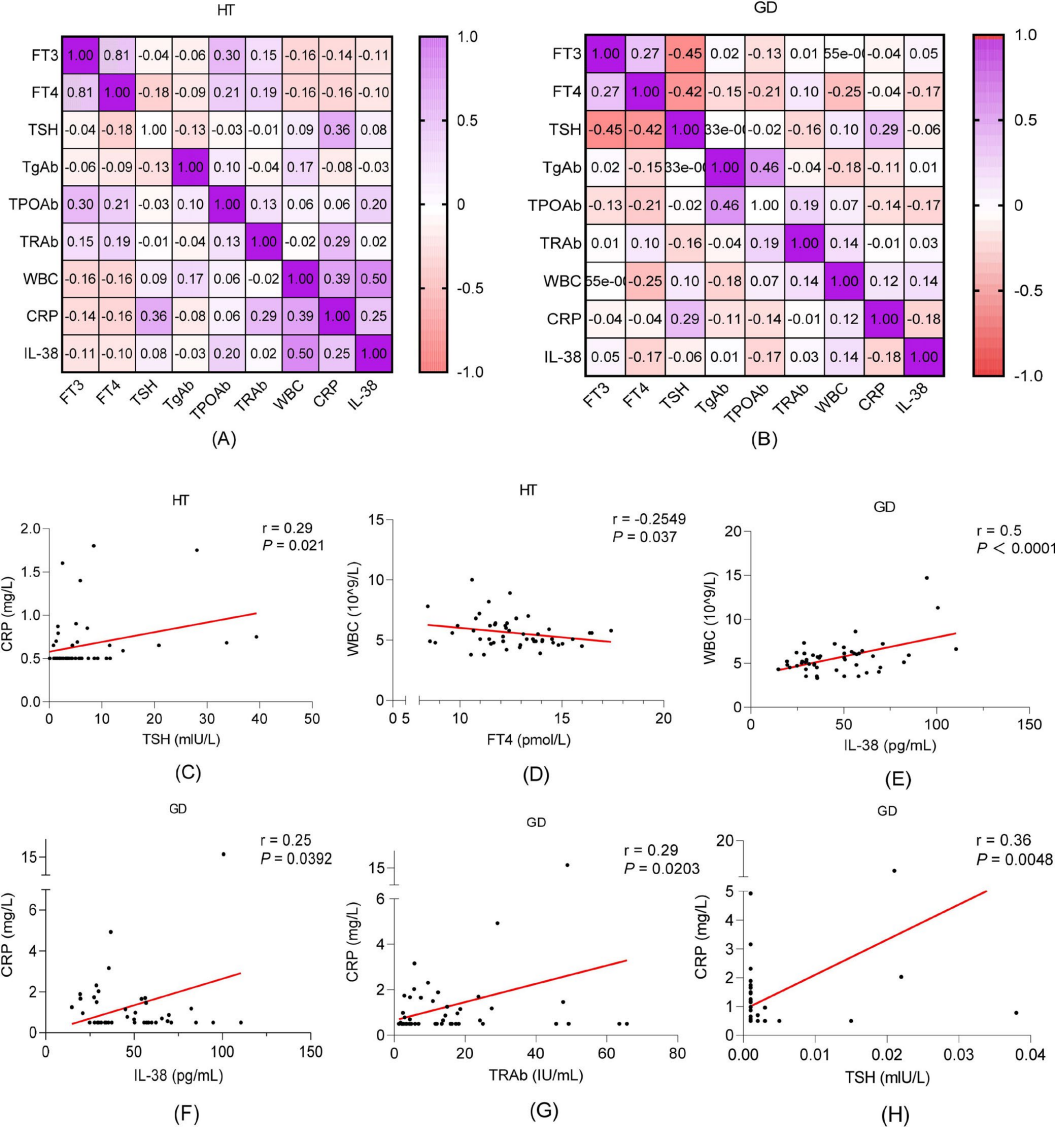
Correlation analysis between serum IL‐38 concentrations, inflammatory markers, and thyroid‐related parameters

We constructed a visual correlation heat map between serum IL‐38 concentrations, inflammatory markers, and thyroid‐related parameters (Figure 3A,B). On the basis of the correlation heat map, Pearson correlation analysis was performed separately for the higher correlation coefficients between IL‐38, CRP, WBC, and thyroid‐related parameters (Figure 3C–H). We found a positive correlation between CRP and TSH concentrations (*r* = 0.29, *p* < 0.05) and a negative correlation between the WBC count and FT4 concentrations (*r* = −0.25, *p* < 0.05) in the HT group. In the GD group, serum IL‐38 concentrations were positively correlated with CRP concentrations (*r* = 0.25, *p* < 0.05) and the WBC (*r* = 0.5, *p* < 0.01), and CRP concentrations were positively correlated with TRAb (*r* = 0.29, *p* < 0.05) and TSH concentrations (*r* = 0.36, *p* < 0.01).

### 3.5 Receiver operating characteristic curves for the diagnosis of AITD

**FIGURE 4:**
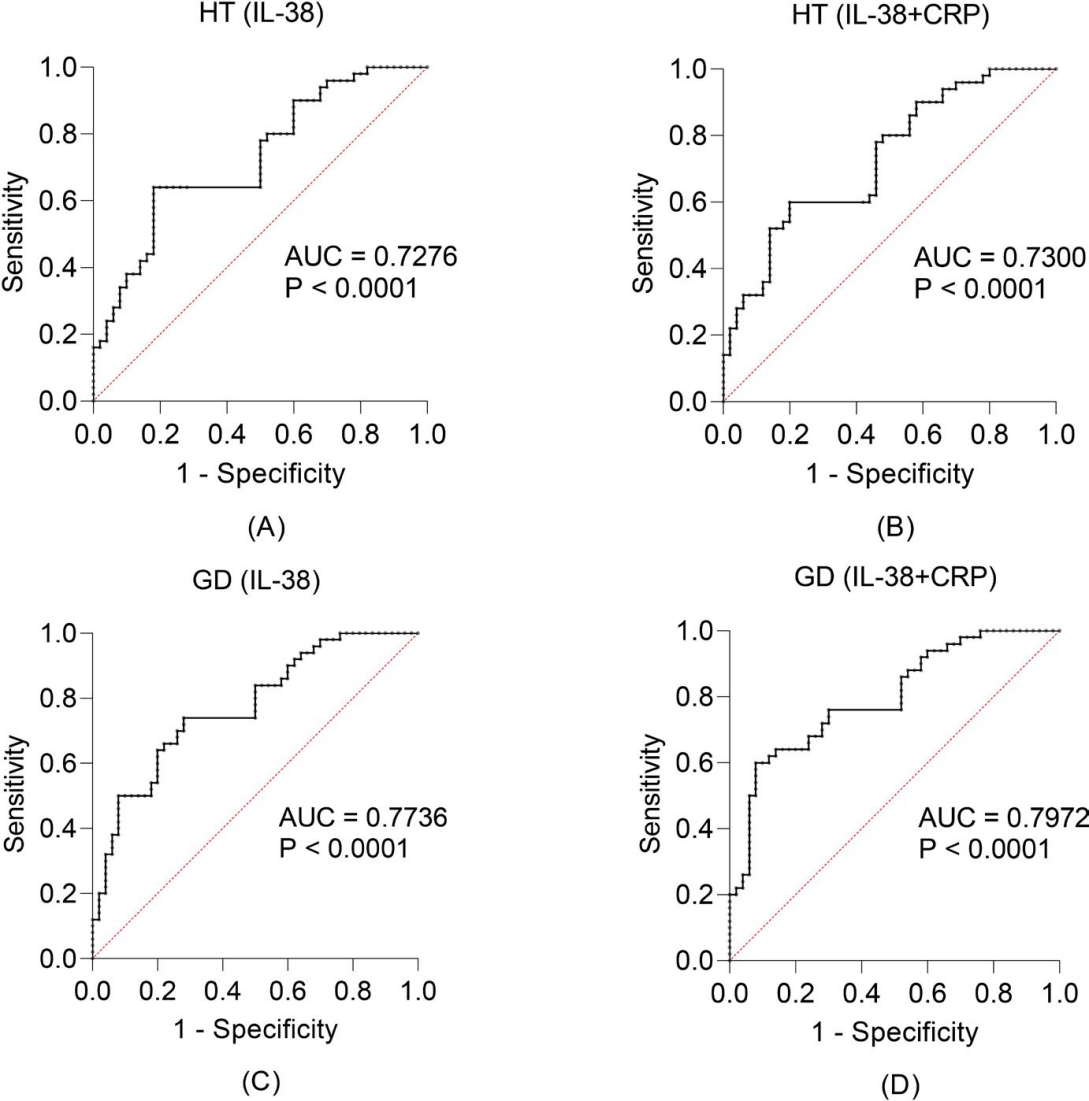
ROC curves for the diagnosis of AITD

We evaluated the diagnostic value of IL‐38 concentrations and IL‐38 combined with CRP concentrations for AITD using receiver operating characteristic (ROC) curves (Figure 4). The area under curve (AUC) was 0.7276 (*p* < 0.01) using IL‐38 concentrations for the diagnosis of HT and 0.7300 (*p* < 0.01) using IL‐38 concentrations in combination with CRP concentrations. The AUC was 0.7736 (*p* < 0.01) when IL‐38 concentrations were used to diagnose GD and 0.7972 (*p* < 0.01) when IL‐38 concentrations were combined with CRP concentrations.

## 4 DISCUSSION

To the best of our knowledge, this is the first study to assess the importance of serum IL‐38 levels for diagnosing AITD. We found that serum IL‐38 concentrations in the GD and HT groups were significantly lower compared with those in the HC group. We also found lower CRP concentrations in the HT group compared with those in the HC group. CRP concentrations tended to be elevated in the GD group compared with the HC group. Principal component analysis showed that serum IL‐38 concentrations, inflammatory indicators, and thyroid‐related parameters were able to distinguish GD and HT from HC. Pearson correlation analysis showed that CRP concentrations were positively correlated with TSH concentrations, and the WBC count was negatively correlated with FT4 concentrations in individuals in the HT group. In the GD group, IL‐38 concentrations were positively correlated with the WBC count and CRP concentrations. CRP concentrations were also positively correlated with TRAb and TSH concentrations in the GD group. ROC curve analysis showed a high diagnostic value of IL‐38 alone and IL‐38 in combination with CRP in GD and HT.

IL‐38 is a new member of the IL‐1 family that can reduce the release of inflammatory mediators by inhibiting inflammatory signaling. IL‐1 receptor 1, IL‐36 receptor, and IL‐1 receptor 10 are potential receptors of IL‐38, and IL‐38 exerts anti‐inflammatory effects by binding to them.^19^, ^20^, ^21^ A growing number of reports have shown that IL‐38 plays an important role in autoimmune diseases. Current research on IL‐38 has focused on RA, SLE, and asthma.^22^, ^23^, ^24^ However, the potential role of IL‐38 in AITD has not been investigated.

In GD and HT, which are autoimmune diseases, there is a disturbance in the balance of inflammatory mediators, such as IL‐35,^5^ IL‐22,^13^ and IL‐17.^11^ Our study showed lower serum IL‐38 concentrations in GD and HT compared with HC, which is consistent with the properties of IL‐38 as an anti‐inflammatory cytokine. However, this finding is in contrast to other reports of high expression of IL‐38 cytokines in inflammatory diseases, such as RA^25^ and SLE.^17^ Maryam et al.^18^ also observed reduced serum IL‐38 concentrations in patients with Behçet's disease. The differences in IL‐38 concentrations in AITD and other autoimmune diseases may be partly attributed to differences in the pathogenesis of the various diseases. Lymphocytic infiltration, autoimmune antibody production, and thyroid‐related hormone dysregulation are the main features of AITD. We found that TPOAb and TgAb concentrations were elevated in patients with GD and HT, while TRAb concentrations were elevated in patients with GD (Table 1). Thyroid‐associated ophthalmopathy (TPO) is an organ‐specific autoimmune disease closely related to GD. Shi et al.^26^ showed that IL‐38 concentrations in the circulation and orbital connective tissue were lower in patients with TPO compared with controls. IL‐38 reduces the production of inflammatory mediators by inhibiting IL‐23R and IL‐17A concentrations in peripheral blood mononuclear cells in patients with TPO *in vitro*.^27^ We also observed lower serum CRP concentrations in patients with HT compared with those in patients with HC. We found that serum CRP concentrations tended to be higher in the GD group than in the HC group. Few studies have reported the relationship between serum CRP concentrations and AITD. Rao et al.^28^ showed that saliva CRP concentrations were lower in patients with HT than in the normal population, which supports our finding. CRP, which is an indicator of inflammation, is an acute phase response protein produced by the body in response to changes in the internal and external environment. Some studies have shown an association between CRP and autoimmune diseases.^29^

Gradual destruction of thyroid lymphoid follicles by autoantibodies and inflammatory mediators released by lymphocytes are the main cause of hypothyroidism in patients with HT.^9^ Elevated TSH concentrations are one of the main manifestations of clinical hypothyroidism in patients with HT.^30^ In our study, we observed a positive correlation between CRP and TSH concentrations in patients with HT. This finding suggests that CRP is useful for the diagnosis of HT and for the assessment of this disease. Positive correlations between CRP and IL‐38 concentrations, and between TRAb and TSH concentrations were observed in patients with GD. Measurement of IL‐38 and CRP would greatly improve the specificity and sensitivity for diagnosing AITD.

## 5 CONCLUSION

This study shows that serum IL‐38 concentrations are lower in GD and HT compared with the healthy population. Additionally, serum CRP concentrations are significantly lower in HT than in the healthy population and tend to be increase in GD. Furthermore, serum IL‐38 and CRP concentrations have good diagnostic value for HT and GD. Our findings will be helpful for future mechanistic studies and for the prevention and treatment of AITD.

## CONFLICTS OF INTEREST

The authors declared that they have no potential conflicts of interest.

## Data Availability

The datasets used and/or analyzed during the current study are available from the corresponding author on reasonable request.
